# Apps and Digital Resources for Child Neurodevelopment, Mental Health, and Well-Being: Review, Evaluation, and Reflection on Current Resources

**DOI:** 10.2196/58693

**Published:** 2025-01-01

**Authors:** Kelsie Ann Boulton, Makana Hilton, Emilia Sutton, Adam John Guastella

**Affiliations:** 1 Children’s Hospital Westmead Clinical School Faculty of Medicine and Health University of Sydney Sydney Australia; 2 Child Neurodevelopment and Mental Health Team Brain and Mind Centre University of Sydney Sydney Australia; 3 Clinic for Autism and Neurodevelopmental (CAN) Research Brain and Mind Centre University of Sydney Sydney Australia

**Keywords:** digital tools, neurodevelopmental conditions, mental health, digital health, implementation, digital interventions, child neurodevelopment, digital technology, mobile phone

## Abstract

**Background:**

An increase in the prevalence of neurodevelopmental conditions worldwide, alongside resource constraints within clinical services, has led to increased interest in health information technologies, such as apps and digital resources. Digital tools are often viewed as a solution to bridge this divide and to increase supports for families. There is, however, a paucity of research that has evaluated digital health tools, their potential benefits for child neurodevelopment and associated concerns (eg, mental health, well-being), and their benefit for families.

**Objective:**

This study conducted the first review of existing mobile apps and digital resources targeted at supporting the needs of children with developmental concerns or neurodevelopmental conditions.

**Methods:**

We identified 3435 separate resources, of which 112 (43 apps and 69 digital resources) met the criteria. These resources were categorized according to their purpose or target and were then reviewed based on their engagement, information quality, and evidence base using the Adapted Mobile App Rating Scale.

**Results:**

The most common condition of concern targeted by apps and digital resources was autism (19/112, 17% resources), with retrieved resources focusing on supporting challenging behaviors, promoting speech, language, and social development, and providing options for alternative and assistive communication. Other common areas of concern targeted by apps and digital resources included language and communication (16/112, 14.3%) and attention-deficit/hyperactivity disorder (11/112, 9.8%). Results showed that reviewed resources were engaging, with high levels of accessibility and functionality. Resources had various functions, including developmental or behavioral tasks targeted at children, assistive communication support, scheduling support, journaling, and advice, activities, and strategies for parents. The information quality of resources, such as credibility of source and evidence base was, however, mostly low. Apps and digital resources with good credibility and an existing evidence base were largely developed in partnership with research, health, or government institutions, and were rated significantly higher on overall quality compared with apps and digital resources not developed in partnership with such institutions (apps; t_41_=–4.35, *P*<.001; digital resources; t_67_=–4.95, *P*<.001).

**Conclusions:**

The lack of evidence base across resources means that it is extremely difficult to provide recommendations to families with respect to apps or digital resources that may support their needs. Frameworks for the development of new tools are discussed, highlighting the novel approaches required to demonstrate the efficacy of tools for improving outcomes for children and families. Such a framework requires collaboration with multiple stakeholders (software developers, researchers, regulatory bodies, clinicians, children, and families) and engagement across multiple levels of expertise (app development, implementation, and dissemination within services, policy, and clinical regulations), to harness the potential of digital health for improving outcomes and promoting support in child neurodevelopment, which at this juncture remains largely underdeveloped.

## Introduction

In recent years, there has been a reported increase in the prevalence of neurodevelopmental conditions (NDCs) [1,2] and a rising awareness of the importance of early diagnosis and intervention in children with signs of developmental delay [3,4]. This has been matched with a growing number of recommendations and guidelines for multidisciplinary supports provided as early as possible [5-7]. Early identification and evidence-based supports are argued to be crucial for enhancing outcomes in the 1 in 10 children diagnosed with a neurodevelopmental condition [4].

Despite this, there is consistent evidence across nations of the considerable delays and barriers children and families face when seeking assessment [8,9] and support [10,11]. In addition, the wide range of multidisciplinary support needs often experienced by children and caregivers are frequently reported to result in fragmented care, where co-occurring conditions (eg, mental health) are often left unaddressed [12-14]. Caregivers can often report feeling overwhelmed and confused, and comment on the lack of coordination of care and difficulty in obtaining trustworthy, accessible information about supports [15-17].

It is unsurprising, given these resource constraints, that many families turn to health information technologies, such as apps and digital tools, to obtain information to supplement their health care [18,19]. The potential utility of such technologies has been reinforced internationally, with global commissioned reports recommending the integration of digital technologies to facilitate and optimize health care for children and adolescents [20,21]. Digital tools may bridge the divide between existing limited resources and growing demand [22], providing more instant access to supports that may be delivered in a flexible manner. Such tools may also bridge geographical barriers, facilitate access to care for diverse communities, and provide additional opportunities for assessment and feedback to support child and family needs across time [23-25].

Despite enthusiasm for the use of innovative digital solutions [26,27], there is an urgent need to understand the digital tools that currently exist for children with NDCs and their families, the platforms that these tools can be accessed on, and whether these tools are reliable, credible and have an existing evidence base to support their implementation. There is also a need for such evaluations to map existing resources so that supports can be strategically developed where gaps exist. For example, it has previously been shown that, despite the promise of digital tools for mental health, only a minority of apps purported to monitor and manage mental health symptoms have clinically validated evidence of effectiveness [28,29]. More recently, a meta-analysis of the efficacy of smartphone apps for symptoms of depression and anxiety reported overall small effects for symptom amelioration [30]. In addition, while there can be many valuable resources available, there are also many poor sources of information [31-33]. As a result, individuals may instead be influenced by factors such as the star rating of an app, which have been shown to have little relationship with clinical utility [34].

In addition to evaluating digital tools in terms of their evidence base and credibility, it is critical to also evaluate these tools in terms of their engagement, aesthetics, and user quality. Worldwide, there is consistent evidence that poor quality of an app or digital resource is associated with abandonment [35]. In contrast, the design and visual features of apps including an engaging interface, and being intuitive and easy to navigate, all increase engagement and use [36]. To illustrate, digital mental health tools have been shown to have continued uptake as low as 0.1% when rolled out at scale and this low rate is believed to be associated with low appeal and poor app maintenance to promote engagement [37]. This means that the ongoing use of a digital tool, no matter the evidence behind it, is likely to fail without high appearance and usability ratings [23,37]. This may be particularly important for families with NDCs as research shows that personal characteristics moderate the type of apps and digital tools that are used [38]. People with NDCs are more likely to learn from well-structured, easy-to-understand, and clear information, further highlighting the need to evaluate the quality and aesthetics of apps and digital resources [39].

The objective of this study was to review available mobile apps and digital resources that are targeted at supporting the needs of children with developmental concerns or NDCs. The study first sought to conduct a systematic review of all existing mobile apps and digital resources and to categorize them according to their purpose or target. Second, the review then sought to evaluate the information quality, utility, and evidence base for each resource by using the Adapted Mobile App Rating Scale (A-MARS) [40] to demonstrate the quality and evidence base of existing tools.

## Methods

### Design

This review used a stepwise approach in a similar manner to a systematic review. Our approach included a search strategy, assessment against prespecified eligibility criteria, app, and digital resource selection through an initial screening of all identified apps and digital resources, a full review of included apps and digital resources, data extraction and analysis, and quality assessment of included apps and digital resources using a reliable quality assessment tool, the A-MARS [40,41].

### Search Strategy

A search of the main online app stores, iTunes (Apple Inc, Australia) and Google Play (Google Inc, Australia), and a search of digital resources using the Google search engine was conducted from October 2023 to November 2023. To avoid a selection bias from personal Google algorithms, search histories were cleared before each search term as per previous recommendations [42]. Search terms were identified following consultations with researchers with expertise in NDCs, clinicians practicing in neurodevelopmental assessment services, and families with lived experience of NDCs. Search terms were selected based on keywords caregivers of children with developmental concerns or NDCs may use when attempting to access apps and digital resources. The final search terms also demonstrated the best performance in identifying apps and digital resources of interest for this review during preliminary searches. In total, 28 search terms were identified by an expert clinical team based on *DSM-5* (*Diagnostic and Statistical Manual of Mental Disorders* [Fifth Edition]) terms for neurodevelopment and transdiagnostic terms that families might typically use. Search terms included neurodevelopment kids, intellectual disability kids, global developmental delay, communication disorder, language disorder kids, stuttering kids, autism spectrum disorder, autism kids, attention-deficit/hyperactivity disorder (ADHD) kids, ADHD kids, learning disorder kids, motor disorder kids, Tourette’s disorder kids, cerebral palsy kids, dyslexia kids, epilepsy kids, anxious kids, depressed kids, sleep problem kids, attention kids, social problems kids, restricted behaviors kids, repetitive behaviors kids, social skills kids, well-being kids, education kids, emotion kids, cognition kids.

### Eligibility Criteria

To be considered for inclusion in this review, apps, and digital resources were required to meet the following inclusion criteria: (1) contain keywords of the search term in the title or description, (2) have a focus on the needs of children with developmental concerns or NDCs (eg, social skills, well-being, academic skills, or mental health), (3) be free of charge, and (4) be in the English language. To ensure the resources contained relevant and applicable information that was catered to families of children with developmental concerns or NDCs, a variety of exclusion criteria was applied. Apps and resources were excluded if they (1) had no actionable information for families to use (eg, purely game, social media site, news article, research paper, clinic website, or home assessment), (2) if they were selling products (eg, books, materials, or therapy sessions), (3) if the information was not relevant to our target population (eg, app or resource targeted only at adults or included information was overly general), or (4) if they were in unsuitable formats for review with the A-MARS (ie, PDF document, podcast, or video).

### Selection Process

Three authors (MH, ES, and KB) carried out the app store and search engine searches. To balance feasibility and search comprehensiveness, the first 50 resources for each search term were selected from both the app stores and the search engine [43]. A predesigned Microsoft Excel spreadsheet developed for this review was used to enter information about the apps and digital resources. Information entered included the name of the app or digital resource, the name of the app developer or the URL for digital resources, the app store or stores in which the app was available, and the search terms or terms that identified each app or digital resource.

Following removal of duplicates between search terms, 1 rater (MH) conducted an initial screen for eligibility, by reviewing the title, description, and home page of the app or digital resource. Additional screening was conducted on the remaining apps and digital resources to confirm inclusion in the review. This screening was conducted by 2 raters (MH and ES) and involved the installation of the app and a more detailed review of the digital resource, including a review of written information, pictures, and videos. Any disagreements were discussed with a 3rd rater (KB) and resolved by consensus. Reasons for exclusion were recorded.

### Data Extraction

Apps and digital resources identified as eligible in the screening phase were then assessed using the A-MARS [40]. The A-MARS consists of 28 items, developed to assess the quality of mobile apps and e-tools (eg, websites and digital resources). The A-MARS provides an evaluation of app and e-tool quality by grading each app or digital resource on several domains, as described below (Textbox 1). Each item was scored using a 5-point Likert scale (1: inadequate, 2: poor, 3: acceptable, 4: good, and 5: excellent). Supplementary questions within the A-MARS capture information pertaining to the health-related quality of the app or digital resource. Eight scores are calculated for the A-MARS, including the mean score for each domain (ie, engagement, functionality, aesthetics, information, subjective quality, and health-related quality), a mean quality score based on the engagement, functionality, aesthetics and information domains, and a mean total score.

**List of Adapted Mobile App Rating Scale subscales and subscale descriptions.**

Subscale and description

**Engagement (5 items)**

- Is the app or digital resource engaging and interesting for the user? Does it have prompts (eg, send alerts, messages, or reminders)?

**Functionality (4 items)**

- How does the app or digital resource function, does it have a logical flow and design? Is it easy to navigate?

**Aesthetics (3 items)**

- How appealing is the app or digital resource in terms of its overall visual appeal, graphic design, and stylistic consistency?

**Information (6 items)**

- Does the app or digital resource contain high-quality information (eg, text, feedback, measures, or references) from a credible source?

**Subjective quality (4 items)**

- Subjective quality rating of the app or digital resource.

**Health-related quality (6 items)**

- Does the app or digital resource provide access to other resources, strategies linked to the target issue, or the option for real-time tracking?

All apps and digital resources were rated using the A-MARS instrument. Three expert raters conducted this review: (1) a senior research fellow with a PhD in Psychology and 10 years’ experience working with pediatric NDCs and digital health; (2) a senior research assistant with a Master’s degree in Brain and Mind Sciences and 3 years’ experience in working with NDCs and digital tools to support children with NDCs and their families; and (3) a research affiliate with Bachelors’ degrees in Psychology and Medical and Health Sciences and a Postgraduate Diploma in Psychology. Two of these raters were also people with a diagnosed NDC. All raters reviewed the A-MARS in depth before conducting initial pilot ratings. Two apps and 2 digital resources were initially reviewed independently for training purposes. After independently rating the apps and digital resources, the raters met to compare and review results and to resolve discrepancies in ratings. To reach a consensus, the raters reviewed the A-MARS in depth to improve the alignment of ratings. The remaining apps and digital resources were then rated independently by 2 raters. Based on the methodology described by others [40,41], each rater trialed the apps and digital resources for a minimum of 10 minutes and then independently rated their quality using the A-MARS.

### Statistical Analyses

Statistical analyses were conducted using SPSS (version 28; IBM Corp). Descriptive statistics were used to summarize the A-MARS domain scores, the mean quality score, and the mean total score across apps and digital resources. Cronbach α and intraclass correlation coefficients were used to calculate the internal consistency and interrater reliability of the A-MARS domain scores, the quality score, and the total score. As this is the first study to our knowledge that has applied the A-MARS in apps and digital resources targeted at child development and NDCs, 2-tailed independent samples *t* tests were also used to compare mean domain scores, quality scores, and total scores between apps and digital resources. Bonferroni corrections were used for these comparisons and a *P* value of <.006 was considered statistically significant. To understand how the involvement of a reputable and credible organization impacted ratings for apps and digital resources, 2-tailed independent samples *t* tests were used to compare domain scores, quality scores, and total scores for apps and digital resources that had been developed in partnership with a university, health or government institution, relative to those that had not been developed in partnership with such institutions.

## Results

### Overview

**Figure 1:**
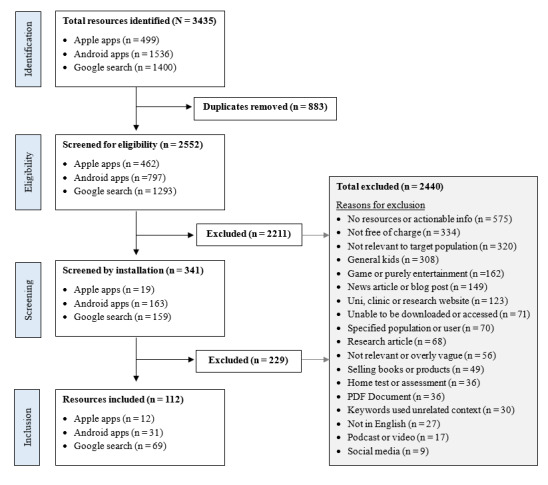
Flowchart of selection of included resources.

The process of identification and inclusion of apps and digital resources is outlined in Figure 1. A total of 3435 apps and digital resources were initially identified. After the removal of duplicates, 2211 apps and digital resources were excluded following initial screening by title and description, and an additional 229 apps and digital resources were excluded following installation and more detailed screening. The most common reason for exclusion was an absence of actionable information that children and families could use (575/2440, 23.6%), followed by not being free of charge (334/2440, 13.6%), and not being relevant to the target population (320/2440, 13.1%). A total of 12 Apple apps, 31 Android apps, and 69 digital resources were included in the final review of this paper.

### Features of Included Apps and Digital Resources

Table 1 displays the primary area of focus for retrieved apps and digital resources. The most common conditions that the resources targeted were autism, language, or communication difficulties, and ADHD. Both apps and digital resources were retrieved for most searched terms; however, resources retrieved for anxiety, attention, and developmental delay were mainly digital resources. Comparatively, the resources retrieved for epilepsy, cerebral palsy, and child well-being were mainly apps. Retrieved apps and digital resources had a range of functions, including developmental or behavioral tasks targeted at children, assistive communication support, scheduling support, journaling, and advice, activities, and strategies for parents.

**Table 1:** Primary focus areas included apps and digital resources.

Topic area	Apps, n (%)	Digital resources, n (%)	Apps and digital resources combined, n (%)
Autism	5 (11.6)	14 (20.3)	19 (17.0)
Language and communication	7 (16.3)	9 (13.0)	16 (14.3)
ADHD^a^	6 (14.0)	5 (7.2)	11 (9.8)
Anxiety	1 (2.3)	9 (13.0)	10 (8.9)
Dyslexia	3 (7.0)	6 (8.7)	9 (8.0)
Mood and depression	2 (4.7)	5 (7.2)	7 (6.3)
Social skills	2 (4.7)	4 (5.8)	6 (5.4)
Emotions	2 (4.7)	4 (5.8)	6 (5.4)
Intellectual disability	3 (7.0)	2 (2.9)	5 (4.5)
Epilepsy	5 (11.6)	0 (0)	5 (4.5)
Attention	1 (2.3)	4 (5.8)	5 (4.5)
Sleep	2 (4.7)	2 (2.9)	4 (3.6)
Cerebral palsy	2 (4.7)	1 (1.4)	3 (2.7)
Developmental delay	0 (0)	3 (4.3)	3 (2.7)
Child well-being	2 (4.7)	0 (0)	2 (1.8)
Tourette	0 (0)	1 (1.4)	1 (0.9)

^a^ADHD: attention deficit hyperactivity disorder.

### Overall Assessment of Apps and Digital Resources

Across the 43 apps and 69 digital resources, excellent internal consistency and inter-rater reliability was found across the total and sub-domain A-MARS scales. Detailed item and domain statistics for the reviewed apps are displayed in Tables S1 and S2 in Multimedia Appendix 1. Table 2 displays the mean and median scores of the reviewed apps (n=43) and digital resources (n=69) across the 5 A-MARS domains, as well as for the quality and total scores. The apps, taken together, scored higher than the minimum acceptable score of 3.0 across most A-MARS domains, excluding the health-related quality domain (Table 2). Apps typically received low scores on this domain due to a lack of additional resources available (mean 1.81, SD=1.22), and limited access to further support or related information (mean 1.85, SD=1.24). Furthermore, the digital resources scored higher than 3.0 across all A-MARS domains except the subjective quality domain (Table 2).

**Table 2:** Adapted Mobile App Rating Scale ratings for apps and digital resources.

Apps					Digital resources				Values	
Domain	Mean (SD)	Median (range)	N (%) scoring ≥3.0	Mean (SD)		Median (range)	N (%) scoring ≥3.0	T-statistic (*df*)		*P* value
Engagement	3.67 (0.74)	3.60 (2.40-4.90)	36 (83.7%)	3.19 (0.54)		3.13 (2.00-4.50)	45 (65.2%)	3.97 (110)		<.001
Functionality	4.13 (0.68)	4.25 (2.25-5.00)	40 (93.0%)	4.09 (0.55)		4.13 (2.50-5.00)	66 (95.7%)	0.37 (110)		.72
Aesthetics	3.73 (0.72)	4.00 (1.84-5.00)	38 (88.4%)	3.66 (0.68)		3.67 (2.00-5.00)	62 (89.9%)	0.56 (110)		.57
Information	3.06 (0.95)	3.13 (1.00-4.75)	27 (62.8%)	3.57 (0.59)		3.67 (2.33-4.75)	61 (88.4%)	-3.16 (110)		.002
Subjective quality	3.21 (0.88)	3.50 (1.50-4.88)	26 (60.5%)	2.89 (0.68)		2.88 (1.38-4.25)	27 (39.1%)	2.04 (110)		.04
Health-related quality	2.58 (0.91)	2.33 (1.00-4.83)	13 (30.2%)	3.34 (0.78)		3.33 (2.00-5.00)	45 (65.2%)	-4.74 (110)		<.001
Quality score	3.40 (0.67)	3.40 (2.17-4.88)	32 (74.4%)	3.62 (0.53)		3.66 (2.38-4.69)	60 (87.0%)	–2.01 (110)		.04
Total score	3.40 (0.64)	3.29 (2.00-4.72)	31 (72.1%)	3.48 (0.55)		3.50 (2.32-4.67)	54 (78.3%)	–0.73 (110)		.47

Both apps and digital resources scored higher than 3.0 across quality and total scores. Apps and digital resources scored high on the functionality domain, with consistently high average scores across subcriteria. Of note, included apps scored relatively low in the information quality domain primarily due to questionable source credibility (mean 2.52, SD 0.79). Furthermore, a minority of apps (3/43, 7%) and no digital resources met the criteria for a verifiable evidence base as outlined by the A-MARS (has been trialed or tested and published in scientific literature). Independent sample *t* tests revealed statistically significant differences in mean scores between apps and digital resources on the engagement, information, and health-related quality domains (*P*<.006). While apps had higher ratings relative to digital resources for engagement, digital resources had higher ratings relative to apps on information and health-related quality domains.

Overall, apps and digital resources that had been developed in partnership with a health, university, or government institution, or where such an institution was listed as a partner, were rated more highly across domains of engagement, functionality, information, subjective quality, and health-related quality, as well as on overall quality and total scores. As shown in Tables 3 and 4, these effects were moderate to large for most comparisons. In total, 19% (8/43) of apps were developed in partnership with a health, university, or government institution, and 48% (33/69) of digital resources were developed in partnership with these institutions.

**Table 3:** Adapted Mobile App Rating Scale ratings for apps split by partnerships with health, university, or government institutions.

Domain	Apps				
	Institution partnership (n=8), mean (SD)	No institution partnership (n=35), mean (SD)	Effect size	T-statistic (*df*)	*P* value
Engagement	4.23 (0.75)	3.54 (0.68)	0.69	–2.53 (41)	.02
Functionality	4.64 (0.30)	4.01 (0.69)	0.64	–2.53 (41)	.02
Aesthetics	4.15 (0.62)	3.64 (0.73)	0.70	–1.85 (41)	.07
Information	4.30 (0.42)	2.78 (0.80)	0.75	–7.52 (41)	<.001
Subjective quality	4.05 (0.58)	3.02 (0.83)	0.80	–3.29 (41)	.002
Health-related quality	3.53 (1.33)	2.36 (0.63)	0.79	–2.42 (41)	.04
Quality score	4.13 (0.58)	3.23 (0.57)	0.57	–4.04 (41)	<.001
Total score	4.14 (0.51)	3.23 (0.54)	0.54	–4.35 (41)	<.001

**Table 4:** Adapted Mobile App Rating Scale ratings for digital resources split by partnerships with health, university, or government institutions.

Domain	Digital resources				
	Institution partnership (n=33), mean (SD)	No institution partnership (n=36), mean (SD)	Effect size	T-statistic (*df*)	*P* value
					
Engagement	3.47 (0.51)	2.93 (0.44)	0.47	–4.76 (67)	<.001
Functionality	4.36 (0.46)	3.83 (0.51)	0.48	–4.58 (67)	<.001
Aesthetics	3.98 (0.62)	3.36 (0.59)	0.6	–4.24 (67)	<.001
Information	3.87 (0.53)	3.30 (0.50)	0.51	–4.60 (67)	<.001
Subjective quality	3.13 (0.70)	2.67 (0.58)	0.64	–2.95 (67)	.004
Health-related quality	3.69 (0.76)	3.02 (0.65)	0.7	–3.95 (67)	<.001
Quality score	3.92 (0.44)	3.35 (0.44)	0.45	–5.18 (67)	<.001
Total score	3.77 (0.50)	3.21 (0.45)	0.47	–4.95 (67)	<.001

### Individual Assessment of Apps and Digital Resources

**Figure 2:**
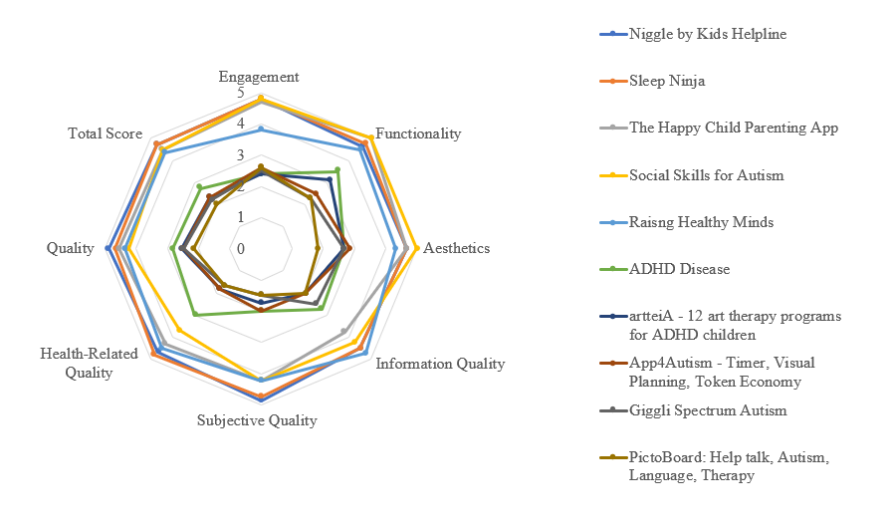
Radar chart of the top 5 and bottom 5 ranked apps. ADHD: attention-deficit/hyperactivity disorder.

**Figure 3:**
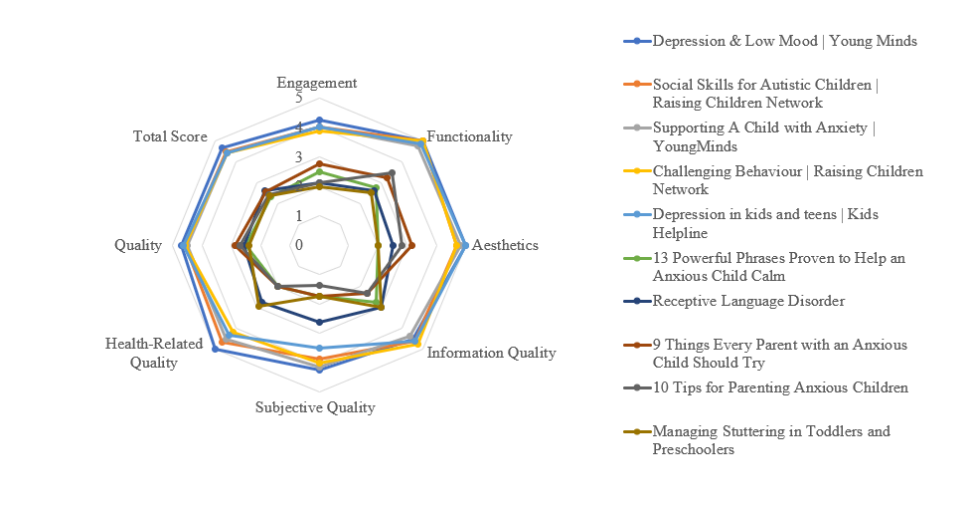
Radar chart of the top 5 and bottom 5 ranked digital resources.

Figures 2 and 3 display scores of the top 5 and bottom 5 apps in terms of their A-MARS ratings. These radar charts show how individual apps and digital resources scored on each A-MARS domain, as well as on the overall quality and total scores. Showing the difference between the top 5 and bottom 5 highlights the disparity between apps and digital resources on A-MARS ratings. In general, the top 5 apps and digital resources were characterized by high levels of factually correct information, relevant resources, good visuals, and engaging and user-friendly interfaces. Highly rated apps and digital resources were developed in collaboration with research, health, or government institutions. In contrast, the bottom 5 apps and digital resources were characterized by less involvement with a reputable institution, defective or inactive links, difficult or cumbersome interfaces, and a lack of information relevant to the purported concern or condition.

## Discussion

This study reviewed existing apps and digital resources that are targeted at the needs of children with developmental concerns or NDCs. To our knowledge, this is the first study that has evaluated apps and digital resources for child neurodevelopment and mental health broadly. From the initial 3435 resources identified, the study reviewed 112 resources in total (43 apps and 69 digital resources). Developed for children with NDCs and their families, the most common resources focus on supports for autism, language or communication difficulties, and ADHD. Most apps and digital resources exceeded the minimum acceptable score of 3.0, suggesting that retrieved resources were engaging, with high levels of accessibility and functionality. However, the results also showed items relating to information quality, such as credibility of source and evidence base, were generally low. Only 7% of apps or digital resources had an established evidence base. Interestingly, mobile apps and digital resources that were developed in partnership with a health, university, or government institution were rated significantly higher across most domains in comparison to apps and resources that were not developed in partnership with such an organization. Unfortunately, the review showed there was almost no evidence for their efficacy or effectiveness in promoting supportive behaviors or improving outcomes. As a result, there is a significant existing divide between the availability of digital tools that can be integrated into family systems to support needs and evidence that warrants recommendations from clinical teams. While the findings of the review provide an evaluation and collation of existing tools, this conclusion highlights the urgent need to develop new frameworks to test efficacy for improving outcomes and promoting supportive behaviors.

These results show that most apps and digital resources alike met the minimum acceptable criteria in terms of quality (eg, highly engaging and functional). However, we did observe some differences as a function of resource type (ie, apps or digital resources). We found that apps displayed higher ratings on the domain of engagement, suggesting that the reviewed apps were more interesting, customizable, and entertaining, compared to digital resources. In contrast, reviewed digital resources had higher ratings on domains of information and health-related quality compared with apps. This indicates that the quality and credibility of information, as well as any additional resources and strategies provided, was higher in the reviewed digital resources.

Apps and digital resources were rated highly in terms of their engagement and functionality, but somewhat lower on information quality, particularly with respect to credibility of source and existing evidence base. We observed that apps and digital resources that had been developed by or in collaboration with a research, health, or government institutions were rated significantly more highly on information quality and credibility. This aligns with studies that have used evaluation tools like the Mobile App Rating Scale and A-MARS in other fields [40,44]. However, we note that very few apps or digital resources had a scientific evidence base to support their use, which points to the dearth of evidence evaluating the effectiveness of apps and digital resources [40,45]. This lack of evidence base may relate to the iterative, fast-paced nature of technological development and the linear, incremental approach to clinical science and research-based interventions. With established lags of up to 17 years for research to be translated into clinical practice, software developers may opt to move to large-scale dissemination of their product before developing a rigorous evidence base [24]. However, the consequence of this is an abundance of apps and digital resources that have little to no evidence base and may not be beneficial for consumers [27]. To resolve this issue, a novel framework with investment prioritized for development is critical, whereby the effectiveness of apps and digital resources can be rigorously evaluated in a streamlined and timely manner. Such a framework needs to incorporate the swift evaluation of real-world implementation alongside both the evaluation of resource development and the evaluation of efficacy. It is unlikely to be achieved by applying standard academic approaches or randomized controlled trial methods and may need to incorporate adaptive research designs within implementation science methodologies. Integrated research enabling platforms [46-48] that permit rapid implementation of clinical trials for multiple apps and resources within existing ethics and governance frameworks are also required for swift and sustained translation.

Our results show that the most common condition of concern targeted by apps and digital resources was autism (19/112, 17.0% of all apps and digital resources), with retrieved resources focusing on supporting challenging behaviors, promoting speech, language, and social development, and providing options for alternative and assistive communication. Given the growing prevalence of conditions like autism [1], an increase in the number of health information technologies focused on these conditions is relevant and has the potential to provide children and families with a variety of support tools. However, the proportion of resources targeted at comorbid conditions, such as mental health, was lower (10/112, 8.9% targeted at anxiety and 7/112, 6.3% targeted at mood or depression). Mental ill health is one of the fastest-growing health problems in children and adolescents, particularly in the wake of the COVID-19 pandemic [49]. Moreover, children with existing developmental delays and NDCs are more likely to experience mental health conditions relative to their neurotypical peers [12]. Together these findings point to a gap in existing digital resources for children with co-occurring developmental and mental health concerns.

It is unlikely that a single resource will meet the varied needs of children and families. The application and evaluation of digital resources to uplift support in the community may require integrated approaches targeting different needs at different stages of child development and different stages of support seeking. Recent arguments have also been made for the integration of digital navigators that can further facilitate the engagement and implementation of digital health tools within multidisciplinary care teams and standard clinical care [40,50]. With the increasing frequency of digital navigators in other disciplines, such as mental health [51,52], health systems providing child development and assessment services can benefit from adopting a similar approach, to support children and families in accessing reliable, credible, and efficacious digital tools across their health care journey.

While this study provided a first review of apps and digital resources targeted at child neurodevelopment, mental health, and well-being, we note certain limitations. The evaluation of apps and digital resources can be made difficult due to the growing number of app evaluation frameworks [50]. While the evaluation tool we selected, the A-MARS, has sound reliability [53], has been used across disciplines [54,55], and has been adapted for use specifically with digital resources [40], we note that there are challenges common across evaluation tools [50]. While it was deemed the most suitable evaluation tool for this review, we note specific limitations of the A-MARS, such as potential subjectivity in ratings, a relative lack of focus on the clinical effectiveness of health information, and a lack of validation across cultures and languages, which may warrant further evaluation of apps and digital resources with additional evaluation tools. However, there is currently no consensus for the evaluation and regulation of apps and digital resources, making it difficult to conduct standardized evaluations that can provide clear guidance and recommendations to patients and clinicians. We also note that we restricted our search to apps and digital resources that were available free of charge. While this may have excluded potentially valuable paid resources, we argue that reviewing freely available resources increases the value of this study for use in the broader community and serves to increase equity in access to supports and resources. In a similar vein, we restricted our search to English language apps and digital resources, potentially limiting the applicability of our findings for non–English speaking populations. However, we have detailed a process that future researchers can use to identify and evaluate apps and digital resources in their region of interest, thereby facilitating evaluations in different countries and contexts. Further, we included a large number of search terms, which resulted in many retrieved resources. As a team we discussed restricting the search strategy; however, it was noted that children with developmental concerns or NDCs often experience multiple transdiagnostic concerns, with caregivers likely to search for support across many of these domains. As such, our more extensive search strategy was considered appropriate in order to capture the broad needs and concerns experienced by this patient population.

A crucial next step for this field is to understand how accessible, reliable, evidence-based health information technologies that meet the needs of children, families, and health care providers can be developed and disseminated across clinical settings. This requires collaboration with multiple stakeholders (app developers, researchers, regulatory bodies, clinicians, children, and families) as well as a reconceptualization of how to implement and recommend health information technologies across clinical services for maximum uptake and maintained engagement. At the development level, for example, partnerships between industry, research, and end-users are key to drive the timely co-production of evidence-based, accessible digital tools. Furthermore, given findings from digital mental health [56], a focus on developing tools that include gamification features may be key to optimizing uptake and engagement by children and families. At the implementation and dissemination level, existing models of care may need to be rethought to optimally engage recipients (ie, patients and clinicians) and encourage the integration of digital tools and solutions into clinical practice. Meanwhile, at the policy level, clinical regulation and recommendations require flexibility to enable innovative digital solutions to be adopted and embedded within health services and systems. While complex and multifaceted, such a framework would enable the development of evidence-based digital tools that are primed for accessibility and engagement with children and families and would facilitate the embedding of digital health into clinical service settings in a sustainable manner, uplifting the capacity of services to provide access to reliable, evidence-based digital tools that are likely to be used to support children and families.

In the current technology-driven world, apps and digital resources are being increasingly used and promoted as a source of information or an adjunct to support and therapy. In this review, we found that a large number of apps and digital resources targeted at child neurodevelopment, mental health, and well-being are engaging and functional, but lack an adequate evidence base. Those apps and digital resources that did show good credibility and an evidence base were largely developed in partnership with research, health, or government institutions. As a result, there is a pressing need to recognize, value, and promote this type of collaboration when developing new digital tools, and to also develop a framework where new digital tools can be rigorously evaluated in a timely manner, to promote swift translation from evidence-based research and development into practice. Clinicians and health care professionals can then be supported to recommend reliable, evidence-based apps and digital resources to children and families based on their individual needs, providing an ideal opportunity to evaluate the effectiveness of these tools for enhancing outcomes.

## Data Availability

The datasets generated during and analyzed during this study are available from the corresponding author on reasonable request.

## Appendix

Multimedia Appendix 1 Interrater reliability and internal consistency of the A-MARS (Adapted Mobile App Rating Scale) items and domain scores.

Multimedia Appendix 2 PRISMA Checklist.

## Abbreviations

ADHD: attention-deficit/hyperactivity disorder
A-MARS: Adapted Mobile App Rating Scale
DSM-5: Diagnostic and Statistical Manual of Mental Disorders (Fifth Edition)
NDC: neurodevelopmental condition
