# Supplementary material for: Apps and Digital Resources for Child Neurodevelopment, Mental Health, and Well-Being: Review, Evaluation, and Reflection on Current Resources
Source: J Med Internet Res. 2025 Jan 1;27:e58693. doi: 10.2196/58693 (PMC11736225; doi:10.2196/58693)
Supplement: Multimedia Appendix 1 [file jmir_v27i1e58693_app1.docx]

**Table S1.** Interrater reliability and internal consistency of the A-MARS items and domain scores based on independent ratings for apps.

| **Subscale/Item** | **Corrected Item-total correlation** | **Mean** | **SD** |
| --- | --- | --- | --- |
| **Engagement alpha = 0.830, ICC = 0.892 (95% CI 0.819-0.943)** | | | |
| 1. Entertainment | 0.831 | 3.709 | 0.773 |
| 1. Interest | 0.839 | 3.709 | 0.789 |
| 1. Customisation | 0.947 | 3.570 | 1.132 |
| 1. Interactivity | 0.924 | 3.000 | 1.215 |
| 1. Target group | 0.817 | 4.349 | 0.736 |
| **Functionality alpha = 0.919, ICC = 0.949 (95% CI 0.915-0.973)** | | | |
| 1. Performance | 0.837 | 4.267 | 0.819 |
| 1. Ease of use | 0.630 | 4.151 | 0.686 |
| 1. Navigation | 0.797 | 4.105 | 0.736 |
| 1. Design | 0.789 | 3.988 | 0.768 |
| **Aesthetics alpha = 0.882, ICC = 0.918 (95% CI 0.859-0.957)** | | | |
| 1. Layout | 0.852 | 3.930 | 0.856 |
| 1. Graphics | 0.727 | 3.605 | 0.821 |
| 1. Visual appeal | 0.903 | 3.663 | 0.713 |
| **Information alpha = 0.831, ICC = 0.875 (95% CI 0.793-0.933)** | | | |
| 1. Goals^a^ | 0.894 | 4.077 | 0.886 |
| 1. Quality of information^a^ | 0.822 | 4.200 | 0.677 |
| 1. Quantity of information^a^ | 0.838 | 3.925 | 0.922 |
| 1. Visual information^a^ | 0.324 | 4.191 | 0.602 |
| 1. Credibility of source | 0.897 | 2.523 | 0.786 |
| 1. Evidence base^b^ | ­­– | 4.000 | 0.000 |
| **Subjective Quality alpha = 0.911, ICC = 0.887 (95% CI 0.764-0.947)** | | | |
| 1. Would you recommend this app/e-tool to people who might benefit from it? | 0.848 | 3.733 | 0.972 |
| 1. How many times do you think you would use the app/e-tool in the next 12 months if it was relevant to you? | 0.708 | 3.454 | 0.999 |
| 1. Would you pay for this app/e-tool? | 0.809 | 2.256 | 1.157 |
| 1. What is your overall star stating of the app/e-tool? | 0.805 | 3.407 | 0.826 |
| **Health-Related Quality alpha = 0.864, ICC = 0.831 (95% CI 0.724-0.909)** | | | |
| 1. Additional resources available? | 0.636 | 1.814 | 1.220 |
| 1. Strategies | 0.820 | 2.454 | 1.443 |
| 1. Solutions | 0.933 | 3.140 | 1.320 |
| 1. Multiple health issues/symptoms addressed | 0.965 | 3.093 | 1.211 |
| 1. Real-time tracking | 0.894 | 3.116 | 1.636 |
| 1. Access to help | 0.706 | 1.849 | 1.237 |

^a^Not all apps met criteria to be rated on these items. Specifically, 13 apps (30%) met criteria for question 13; 20 apps (47%) met criteria for both questions 14 and 15, 21 apps (49%) met criteria for question 16.

^b^A corrected item-total correlation score was not calculated for question 18 as only three apps (7%) met criteria to be rated on evidence base.

**Table S2.** Interrater reliability and internal consistency of the A-MARS items and domain scores based on independent ratings for digital resources.

| **Subscale/Item** | **Corrected Item-total correlation** | **Mean** | **SD** |
| --- | --- | --- | --- |
| **Engagement alpha = 0.791, ICC = 0.581 (95% CI 0.293-0.754)** | | | |
| 1. Entertainment | 0.753 | 3.544 | 0.701 |
| 1. Interest | 0.723 | 3.536 | 0.666 |
| 1. Customisation | 0.715 | 1.478 | 0.735 |
| 1. Interactivity^a^ | - | - | - |
| 1. Target group | 0.720 | 4.189 | 0.670 |
| **Functionality alpha = 0.829, ICC = 0.823 (95% CI 0.716-0.890)** | | | |
| 1. Performance | 0.554 | 4.725 | 0.433 |
| 1. Ease of use | 0.663 | 3.949 | 0.733 |
| 1. Navigation | 0.590 | 4.022 | 0.597 |
| 1. Gestural design | 0.783 | 3.645 | 0.866 |
| **Aesthetics alpha = 0.872, ICC = 0.891 (95% CI 0.840-0.928)** | | | |
| 1. Layout | 0.744 | 3.920 | 0.736 |
| 1. Graphics | 0.615 | 3.478 | 0.715 |
| 1. Visual appeal | 0.767 | 3.573 | 0.819 |
| **Information alpha = 0.838, ICC = 0.636 (95% CI 0.410-0.777)** | | | |
| 1. Goals^a^ | - | - | - |
| 1. Quality of information | 0.731 | 3.848 | 0.693 |
| 1. Quantity of information | 0.816 | 3.725 | 0.851 |
| 1. Visual information | 0.600 | 4.225 | 0.595 |
| 1. Credibility of source | 0.798 | 3.073 | 0.495 |
| 1. Evidence base^a^ | - | - | - |
| **Subjective Quality alpha = 0.887, ICC = 0.747 (95% CI 0.505-0.863)** | | | |
| 1. Would you recommend this app/e-tool to people who might benefit from it? | 0.779 | 3.928 | 0.880 |
| 1. How many times do you think you would use the app/e-tool in the next 12 months if it was relevant to you? | 0.716 | 2.732 | 0.591 |
| 1. Would you pay for this app/e-tool? | 0.575 | 1.609 | 0.844 |
| 1. What is your overall star stating of the app/e-tool? | 0.796 | 2.913 | 1.237 |
| **Health-Related Quality alpha = 0.772, ICC = 0.819 (95% CI 0.737-0.880)** | | | |
| 1. Additional resources available? | 0.825 | 2.913 | 1.237 |
| 1. Strategies | 0.690 | 3.899 | 0.942 |
| 1. Solutions | 0.624 | 4.333 | 0.852 |
| 1. Multiple health issues/symptoms addressed | 0.591 | 3.522 | 0.917 |
| 1. Real-time tracking | 0.781 | 2.565 | 1.440 |
| 1. Access to help | 0.810 | 2.819 | 1.301 |

^a^These items were not included in the domain and total scores for digital resources, as many of the retrieved digital resources did not meet criteria for evaluation on these items.
